# Long-term effects of pallidal deep brain stimulation in tardive dystonia: a follow-up of 5–14 years

**DOI:** 10.1007/s00415-022-10965-8

**Published:** 2022-01-27

**Authors:** Patricia Krause, Daniel Kroneberg, Doreen Gruber, Kristin Koch, Gerd-Helge Schneider, Andrea A. Kühn

**Affiliations:** 1 Movement Disorder and Neuromodulation Unit, Department of Neurology, Charité, University Medicine Berlin, Campus Mitte, Charitéplatz 1, 10117 Berlin, Germany; 2 Department of Neurology and Stereotactic Surgery, University Medicine of Magdeburg, Magdeburg, Germany; 3 Department of Psychiatry and Psychotherapy, Charité, University Medicine Berlin, Campus Mitte, Berlin, Germany; 4 Department of Neurosurgery, Charité, University Medicine Berlin, University Medicine Berlin, Campus Mitte, Berlin, Germany

**Keywords:** Dystonia, Tardive, Pallidal DBS, Long-term effects, DBS and quality of life

## Abstract

**Introduction:**

Pallidal DBS is an established treatment for severe isolated dystonia. However, its use in disabling and treatment-refractory tardive syndromes (TS) including tardive dyskinesia and tardive dystonia (TD) is less well investigated and long-term data remain sparse. This observational study evaluates long-term effects of deep brain stimulation (DBS) of the globus pallidus internus (GPi) in patients with medically refractory TS.

**Methods:**

We retrospectively analyzed a cohort of seven TD patients with bilateral GPi-DBS. Involuntary movements, dystonia and disability were rated at long-term follow-up (LT-FU) after a mean of 122 ± 33.2 SD months (range 63–171 months) and compared to baseline (BL), short-term (ST-FU; mean 6 ± 2.0 SD months) and 4-year follow-up (4y-FU; mean 45 ± 12.3 SD months) using the Abnormal Involuntary Movement Scale (AIMS) and the Burke–Fahn–Marsden Dystonia Rating Scale (BFMDRS), respectively. Quality of life and mood were evaluated using the SF36 and Beck Depression Index (BDI) questionnaires, respectively.

**Results:**

At LT-FU patients had improved by 73% ± 14.2 SD in involuntary movements and 90% ± 1.0 SD in dystonia. Mood had improved significantly whereas quality of life remained unchanged compared to baseline. No serious long-lasting stimulation-related adverse events (AEs) were observed. Three patients of this cohort presented without active stimulation and ongoing symptom relief at long-term follow-up after 3–10 years of continuous DBS.

**Conclusion:**

Pallidal DBS is a safe and effective long-term TD treatment. Even more interesting, three of our patients could stop stimulation after several years of DBS without serious relapse. Larger studies need to explore the phenomenon of ongoing symptom relief after DBS cessation.

**Supplementary Information:**

The online version contains supplementary material available at 10.1007/s00415-022-10965-8.

## Introduction

Tardive syndromes (TS) encompass a broad spectrum of abnormal involuntary movements (AIMs) of the tongue, jaw, trunk and/or extremities emerging after at least 3 months of exposure to dopamine receptor blocking agents, but also after treatment with certain antiemetics and antidepressants [1]. Clinical presentation of TS varies widely between, e.g. dyskinetic, dystonic, stereotypic, tremulous, choreiform and athetoid movements [1]. Up to 21% of the patients treated with dopamine receptor blocking agents (DRBA) are estimated to develop tardive symptoms [1]. TS is often associated with stigmatization and incapacity causing socioemotional distress leading to increased mortality and morbidity [2]. Treatment of TS is challenging and often disappointing. First and foremost, causative drugs ought to be avoided. Other medical therapeutic regimen include dosage reduction, substitution of atypical neuroleptics and the probatory use of tetrabenazine, anticholinergics, botulinum toxin, amantadine, benzodiazepines, propranolol or antioxidants [1]. A recent review reported that more than 50% of TS cases were irreversible after withdrawal from the responsible neuroleptics [3]. The remission rate of TS is yet unclear ranging between 2 and 12% after up to 4 years of discontinuation or reduction of mostly DRBA [1]. Deep brain stimulation (DBS) of the globus pallidus internus (GPi) is an effective treatment for medically refractory dystonia and has progressively evolved into a widely available therapeutic strategy in dystonia as it reduces not only motor impairment but also disability [4]. However, compared to isolated dystonia [5], its use in disabling and treatment refractory TS including tardive dyskinesia and tardive dystonia (TD) is less well investigated. According to the most recent reviews, 24 single case reports and 6 rather small open-label case series reported GPi-DBS to be a safe and promising treatment option, with improvements between 30 and 90% on disease-specific scales after up to 7 years [6–8]. Among the three controlled studies [9–11], the only randomized, sham-controlled trial using a delayed-start design of pallidal neurostimulation in TD did not reach a significant difference between sham and active stimulation in the blinded, controlled phase [10]. At 6-month open follow-up, however, the study cohort showed a mean 40% improvement of dystonia along with improvements in quality of life (QoL) [10]. Similarly, the French STARDY group reported 50% motor improvement in 10 patients after 6 months of GPi-DBS [9]. The longest follow-up, so far, has been reported in 14 TD patients after 6–11 years of GPi-DBS with an overall 63 and 58% motor improvement measured by the AIMS and extrapyramidal symptom rating scale, respectively [11]. Long-term outcome as well as information on QoL, mood and side effects of this potentially life-long therapy are of special clinical interest.

## Methods

### Patients

Seven TD-patients (six women) with pallidal DBS operated between 2001 and 2008 at the University Hospital Charité, Berlin, Germany, were available for long-term follow-up after 5 to 14 years of stimulation. Patients had a mean disease duration of 65.2 ± 48.4 months (mean ± standard deviation; range 12–132 months) and a mean age at surgery of 57.6 ± 17.4 years (range 30–75 years). TD was either attributed to prior antipsychotic or antiemetic treatment. None of the patients responded to various medical treatment attempts (see Suppl. Table). Long-term follow-up presented here (LT-FU; mean 121.7 ± 33.2 months; range 63–171 months) includes assessment of motor impairment, mood, QoL, cognition, stimulation parameters, DBS side effects, medication (see Table 1 and Suppl. Table 1). These data were compared retrospectively to data from baseline (BL; preoperative), short-term follow-up (ST-FU; mean 6 ± 1.9 months; range 4–9 months) and 4-year follow-up (4y-FU; mean 44.9 ± 12.3 months; range 26–65 months) that were all video documented [10, 12]. The study was approved by the local ethics committee. All patients gave their written informed consent.

**Table 1:** Individual patient scores and mean data of involuntary movements (AIMS), dystonia and disability (BFMDRS-M/D) at BL, ST-FU, 4y-FU and LT-FU as well as individual ratings in mood and cognition validated by the BDI and MMSE in patients 1–5 or HAM-D and Mattis in patients 6 and 7 at BL and LT-FU (mean data given only for patients 1–5)

Patient	LT-FU (years)	AIMS BL	AIMS ST-FU	AIMS 4y-FU	AIMS LT-FU	BFMDRS (M/D) ST-FU	BFMDRS (M/D) 4y-FU	BFMDRS (M/D) LT-FU	BDI or HAM-D^a^ (BL/LT-FU)	MMSE or Mattis^b^ (BL/LT-FU)
1	14	19	3	4	5	8/3	9/1	4/1	18/5	29/28
2	12	20	3	4	5	2/3	3/3	3/1	12/3	27/28
3	12	33	6	0	5	15/0	0/0	4.5/1	3/3	28/28
4	10	15	11	10	8	16/5	13/8	3.5/3	21/5	27/28
5	10	24	4	5	4	6/0	5/3	2.5/0	20/11	27/25
6	8	11	0	5	4	4/1	4.5/0	4.5/0	13^a^/0^a^	140^b^/142^b^
7	5	25	1	0	5	0/0	4/0	2/0	17^a^/7^a^	144^b^/144^b^
Mean ± SD	10 ± 3.0	22 ± 8.7	4 ± 3.7	4 ± 3.4	5 ± 1.3	7 ± 6.1/2 ± 2.0	6 ± 4.6/2 ± 2.9	3 ± 1.0/1 ± 1.1	14.8 ± 6.7/5.4 ± 3.0	

*AIMS* Abnormal Involuntary Movement Scale, *BFMDRS* Burke–Fahn–Marsden Dystonia Scale for motor impairment (M) and degree of disability (D), *BDI* Beck depression inventory, *MMSE* Mini-Mental State Exam, *HAM-D* Hamilton depression rating scale, *Mattis* Mattis dementia rating scale, *BL* baseline, *ST-FU* short-term follow-up, *4y-FU* 4 year-follow-up, *LT-FU* long-term follow-up

HAM-D-values are marked with ^a^ and Mattis values are marked with ^b^, respectively

### Clinical assessments

LT-FU assessment of motor symptoms was performed in a non-blinded fashion employing the Abnormal Involuntary Movement Scale (AIMS) for tardive dyskinesia and the Burke–Fahn–Marsden Dystonia Rating Scale (BFMDRS) for dystonia severity and disability [13, 14]. Individual effects of stimulation on health-related QoL were assessed using the SF36 [15]. Occurrence of depressive symptoms was assessed using the Beck Depression Index (BDI) or the Hamilton depression scale (HAM-D) [16, 17]. Cognition was rated by use of Mini-Mental State Exam (MMSE) or Mattis dementia rating scale [18, 19]. All scores were compared to available archival BL and 4y-FU data. All reported device-related side effects and adverse events (AEs) were collected retrospectively from archival records. Patients were additionally asked about chronic side effects or AEs at LT-FU.

### Statistical analysis

Motor function, disability and QoL data were compared between BL and the different FU time points using Friedman test and post hoc Wilcoxon test. A Spearman’s correlation was done to investigate possible correlations between motor outcome and demographic factors such as age at onset, disease severity, changes in QoL and mood (SF-36 and BDI). All data are given as mean ± SD if not mentioned otherwise. A *P* value < 0.05 was considered to be significant.

## Results

**Fig. 1:**
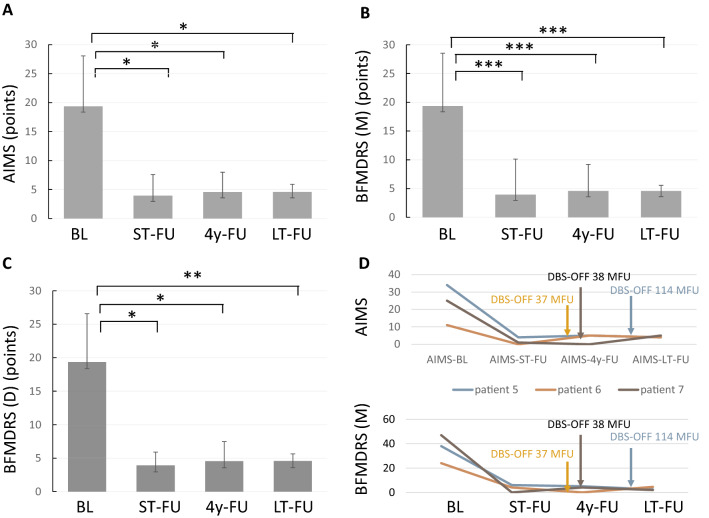
**A** Mean absolute AIMS score at baseline (BL), short-term FU (ST-FU), mean 4-year follow-up (4y-FU) and mean long-term follow-up (LT-FU). **B**, **C** Mean absolute BFMDR motor and disability score at BL, ST-FU, 4y-FU and LT-FU. **(D)** Individual AIMS and BFMDR motor scores for patients 5–7 in whom DBS was switched OFF after more than 3 years of continuous pallidal stimulation. Time points for DBS-OFF at individual follow-up months (MFU) are marked with an arrow. *p < 0.05; **p < 0.005; ***p < 0.005

Mean motor scores were significantly improved at LT-FU compared to preoperative baseline for AIMS and for BFMDRS motor scores leading to a mean improvement of 73 ± 14.2% and 90 ± 1.0%, respectively. BFMDRS disability score improved by 79 ± 1.1% at LT-FU. All patients presented with mild dystonic features before surgery that were predominantly affecting head/neck and upper extremities. Subgroup analysis of the BFMDR motor score revealed a predominant antidystonic effect on the subitems mouth (− 4.8 pts. ± 1.2 at 4y-FU; − 5.2 pts ± 1.5 at LT-FU; p = 0.0002 and 0.0003, respectively) and neck (− 5.1 pts. ± 2.5 at 4y-FU; − 5.7 pts ± 2.4 at LT-FU; p = 0.002 and 0.0008, respectively). The DBS-induced improvement was sustained and consistent over the follow-up period (Fig. 1A–C and Table 1). Importantly, at LT-FU, only patients 1–4 presented with activated pallidal stimulation, whereas patients 5–7 had discontinued stimulation without significant deterioration of TD for 29.3 ± 22.1 months (range 6–59 months; Fig. 1 D). QoL ratings using the SF36 revealed no significant change at LT-FU (59.5 ± 28.27 points) in comparison to BL (53 ± 30.8 points). Sub-analysis of the SF36 physical component score (PCS) versus the SF36 mental component score (MCS) showed a tendency towards higher improvements in the MCS with 53 ± 26 points (range 0–100) at baseline and 62.6 ± 21.9 points (range 0–100) at LT-FU versus 55 ± 36 points (range 0–100) at baseline and 56.5 ± 34 points (range 0–100) in the PCS. However, this difference was not statistically significant. Mean BDI depression score in patients 1–5 were significantly decreased at LT-FU and showed stable reduction in HAM-D in patient 6 + 7 (see Table 1). Cognition remained stable over time (as assessed with MMSE in patients 1–5 and Mattis score in patient 6 + 7; see Table 1). There was no correlation between motor or non-motor improvement with DBS and age of onset or disease duration (data not shown). The main reason for surgical intervention after electrode implantation was IPG replacement after battery exemption with on average 1.6 ± 0.4 IPG replacements (range 1–3) in patients 1–5 over a period of 122 months (range 117–171) and a mean replacement interval of 47.3 ± 7.1 months (range 36–54). Patients 6 and 7 did not reach battery exemption. Patient 6 had the IPG explanted 8 years after implantation due to symptom remission. Four out of seven patients had switched to rechargeable stimulation devices at LT-FU. Since initial implantation, two adverse events (AE) were device related (tension along lead wires) with one being classified as serious adverse events (SAE) requiring surgical intervention due to intolerable pain alongside the lead wires. Patient 4 reported on transient stimulation-related dysarthria and gait disturbances remitting after stimulation parameter adjustments. No long-term side effects such as bradykinesia or gait disturbances were observed in our cohort.

## Discussion

Here we present a retrospective long-term observation of more than 5 years of a patient cohort with severe tardive dystonia and therapeutic bilateral pallidal neurostimulation. The patients in our TD cohort presented with 70% reduction of involuntary movements and about 90% reduction of dystonic symptoms after 5–14 years of pallidal DBS. This excellent outcome is in line with other reports on pallidal DBS in TD with especially good suppression of phasic and/or tremulous dystonic movements [20, 21] whereas larger studies showed smaller DBS effects [10, 11]. While our patient cohort showed predominant phasic or tremolous dystonic movements, the higher variability of clinical responses across studies may be related to clinical subtypes of tardive syndromes. All our patients presented with mild dystonic features at baseline predominantly affecting head, neck, and upper extremities. While several studies reported on equal responses of affected dystonic body regions [11, 22], others found oro-buccal-lingual dystonia to be less responsive than cervical dystonia [23]. Interestingly, our patients demonstrated similar motor effects for oro-buccal-lingual symptoms, axial and limb dystonia after 60–80 months of pallidal stimulation [12] with a predominant amelioration in the subitems ´mouth´ and ´neck´ in the long-term follow-up subgroup analysis. This goes in line with other observations presenting gradual improvements of particular body regions over time requiring more and longer programming sessions to achieve meaningful improvement [24]. All our patients suffered from dystonic symptoms in multiple body parts (see Suppl. Table 2). Patients 5–7 that could stop DBS after several years of continuous treatment initially presented with akathisia and/or high-frequency tremor additionally to their dystonic symptoms (see suppl. video); however, the general clinical pattern of dystonia did not differ in those patients. Nevertheless, patients 5–7 showed persistent remission of symptoms when DBS was stopped after continuous treatment for 10 (patient 5) and 3 (patients 6 and 7) years, respectively. Importantly, those patients had a disease duration of 2, 4, 5 years before surgery and showed a clinically relevant reoccurrence of involuntary movements during OFF testing at short-term follow-up (ST-FU visit). Consequently, it is rather unlikely that the remission reflects the natural course of the disease. While maintenance of benefits after DBS interruption was reported episodically in isolated dystonia [25, 26], several groups have rather reported on reappearance and even rebound of TD after discontinuation of stimulation up to now [11, 27, 28]. In line with this, battery depletion in patient 3 immediately resulted in recurrence of involuntary movements after 11 years of GPi-DBS. Only little is known about spontaneous remission rate in TD. Several groups reported on symptom reduction rather than remission in less than 13% of the patients within 2 to 4 years of discontinuation of the triggering pharmaceutical [29–31]. Prior medication, underlying disease, age at onset, age at surgery or stimulation parameters did not differ between stimulated and non-stimulated patients at LT-FU. The OFF-subgroup had already suffered from TD for 2–7 years before surgery and experienced another 3–10 years of continuous DBS before they were switched off permanently. Consequently, it is rather unlikely that the remission reflects the natural course of the disease. Interestingly, the initial clinical presentation in patients 5–7 was dominated by akathisia and/or highly frequent tremor additionally to dystonic features and variable oromandibular symptoms (see suppl. video). In contrast, generalized dystonic features dominated the clinical picture in patients 1–4. As we cannot but speculate if DBS may induce long-term neuroplastic changes in specific subtypes of TD, this needs to be explored in larger cohorts and by means of complementary electrophysiological and image-based pre- versus post-interventional approaches in clinically well-characterized patients.

Gruber et al. found a significant 46% improvement limited to the physical health SF36 subscore and a trend towards improvements in all other subscores at 4-yr FU in the initial cohort, which includes patients 1–5 of the present cohort [12]. Our patients showed a tendency towards higher improvements in the mental component subscore of the SF36 at long-term follow-up. Overall SF36 scores at LT-FU, however, showed no significant change compared to baseline. One has to keep in mind that the mean age of the total cohort was meanwhile ~ 70 years, and patients suffered from several additional comorbidities with potential impact on QoL. Interestingly, we observed a sustained improvement of depressive symptoms in our patients at LT-FU and no psychiatric adverse events. Psychiatric preconditions traditionally represent a relative contraindication for neurostimulation in DBS studies. No psychiatric adverse events occurred in our patients, which supports the conclusion of a recent review of 117 TD patients with DBS [6] and confirms that pallidal DBS is safe and effective in TD. Nevertheless, we have to consider that our study includes only a small sample size, non-blinded evaluation of stimulation benefits and probable interrater variability due to the retrospective character of the study. However, our study provides a comprehensive assessment of motor features, disability, QoL and mood in a long-term follow-up on an older TD population. Importantly, psychiatric comorbidity was not aggravated by DBS, supporting the recommendation of pallidal DBS in pharmacologically refractory severe TD with psychiatric stable condition. The most interesting observation is the persisting symptom remission in three out of seven patients after 3–10 years of chronic pallidal stimulation.

## Supplementary Information

Below is the link to the electronic supplementary material.

Video Legend: Segment 1: Patient 5 presented with severe and disabling oromandibular dyskinesia interfering with eating, drinking and speaking as well as involuntary movements of mainly the trunk and both legs after therapy with metoclopramide. Following pallidal deep brain stimulation (DBS), involuntary movements had improved meaningfully as early as after 6 months of stimulation. These benefits increased over the course of the following years and remained stable for up to eight years. Because of lacking symptom reappearance after an accidental longer period of DBS OFF-state, it was decided to leave the stimulation switched off. Regular follow-up examinations showed stable symptom suppression and the patient felt no need for reactivation of the DBS. Segment 2: Patient 6 experienced modest oromandibular dyskinesia, dystonic posture of the neck and relevant phasic truncal dystonia interfering especially with stance and gait. Initial benefits were achieved as soon as 6 months after stimulation. At ST-FU, especially dystonic features had reduced significantly with oromandibular dyskinesia, reemerging only slightly under distraction and in discomfort. Because of lacking symptom deterioration after clinical OFF-testing, chronic OFF was tested in 2012 with persisting symptom reduction in the stimulation OFF-state at LT-FU four years later. Segment 3: Patient 7 developed a generalized dystonia with accentuation of the neck and arms, intermittent choreatic movements of the arms as well as tremor of the upper right extremity and involuntary tremulous movements of the tongue interfering with speaking and eating. 6 months after pallidal DBS, symptoms had ameliorated significantly and persistently. During clinical OFF-testing 2.5 years after chronic DBS symptom did not reoccur and DBS remained switched off. Except for minor symptoms such as occasionally slightly increased blink rate of the eyes and discrete involuntary movements of the fingers while walking, symptoms remained in remission until LT-FU 3 years later

Supplementary file2 (DOCX 16 KB)

Supplementary file3 (DOCX 14 KB)
