# Supplementary material for: Long-term effects of pallidal deep brain stimulation in tardive dystonia: a follow-up of 5–14 years
Source: J Neurol. 2022 Jan 27;269(7):3563–8. doi: 10.1007/s00415-022-10965-8 (PMC9217904; doi:10.1007/s00415-022-10965-8)
Supplement: Supplementary file 2 — Supplementary file2 (DOCX 16 KB) [file 415_2022_10965_MOESM2_ESM.docx]

**Suppl. Table 1:**

| **P**  **a**  **t**  **i**  **e**  **n**  **t** | **S**  **e**  **x** | **A**  **A**  **O**  (yrs.) | **Disease Duration before surgery** (months) | **Indication for neuroleptic treatment** | **Predominant symptoms** | **Causing Treatment** | **Medication (pre DBS)** | **Medication**  **(LT-FU)** | **Stimulation Settings**  **ST-FU** | | | | **Stimulation Settings**  **LT-FU** | | | |
| --- | --- | --- | --- | --- | --- | --- | --- | --- | --- | --- | --- | --- | --- | --- | --- | --- |
|  |  |  |  |  |  |  |  |  | **Active**  **contacts**  (right and  left) | **Ampl.**  **(V)**  (right and left) | **Frequ. (Hz)** | **PW (µs)** | **Active**  **contacts**  (right and  left) | **Ampl.**  **(V)**  (right and left) | **Freq.**  **(Hz)** | **PW (µs)** |
| **1** | F | 62 | 48 | Psychosis | phasic dystonia of neck, trunk and right arm | Fluspirilene | Trimipramine  Paroxetine | None | 0-1-  4-5- | 3.2  3.2 | 130 | 60 | 0-1-  4-5- | 3.2  3.2 | 130 | 60 |
| **2** | F | 45 | 132 | Depression | tongue protrusion, phasic dystonia of neck and arms | Haloperidol | Lithium | Lithium | 1-  5- | 1.5  1.5 | 130 | 90 | 1-  5- | 1.4  1.6 | 130 | 90 |
| **3** | M | 27 | 132 | Schizophrenia | generalized phasic dystonia with BSP and mouth contractions | Fluphenazine | Clozapine | Clozapine | 1-2-  5-6- | 2.9  2.8 | 170 | 60 | 1-2-  5-6- | 3.9  1.9 | 170 | 60 |
| **4** | F | 69 | 12 | Depression | orolingual dyskinesia, cervical dystonia, dyskinesia of legs | Fluspirilene | Tetrabenazine | None | 0-  5- | 4  4 | 130 | 90 | 0-1-2+  4-5-6+ | 1  1 | 130 | 90 |
| **5** | F | 73 | 24 | Gastritis | orolingual,  -mandibular dyskinesia,  trunkal dystonia, akathisia legs>hands | Metoclo-  pramide | None | Donepezil | 0-  4- | 2.6  3.5 | 180 | 90 | OFF | OFF | OFF | OFF |
| **6** | F | 65 | 48 | Psychosis | oro-  mandibular dyskinesia, phasic dystonia of trunk, tremulous leg movements | Amisulprid  Olanzapine | Trimipramine,  Mirtazapine,  Clozapine | Citalopram,  Doxepin | 0-  4- | 3.0  2.0 | 130 | 90 | OFF | OFF | OFF | OFF |
| **7** | F | 23 | 60 | Psychosis | tongue tremor, dystonia of face and arms | Haloperidol | Lorazepam,  Clozapine,  Nortriptylin | Clozapine | 1-  5- | 2.5  2.5 | 130 | 90 | OFF | OFF | OFF | OFF |

**Suppl. Table 1:** Demographic patients´ characteristics including psychiatric disease, initial clinical presentation, causing agent of tardive symptoms, pre- and postsurgical medication and individual pallidal stimulation settings at ST-FU and LT-FU. AAO: age of onset; BSP: blepharospasm, ST-FU: short-term follow-up; LT-FU: long-term follow-up; Ampl.: Amplitude; Frequ.: Frequency; PW: pulse width
