# Supplementary material for: Long-term effects of pallidal deep brain stimulation in tardive dystonia: a follow-up of 5–14 years
Source: J Neurol. 2022 Jan 27;269(7):3563–8. doi: 10.1007/s00415-022-10965-8 (PMC9217904; doi:10.1007/s00415-022-10965-8)
Supplement: Supplementary file 3 — Supplementary file3 (DOCX 14 KB) [file 415_2022_10965_MOESM3_ESM.docx]

**Suppl. Table 2:**

| Patient | BFMDRS (BL, 4y- and LT-FU)  **eyes** | BFMDRS (BL, 4y- and LT-FU)  **mouth** | BFMDRS (BL, 4y- and LT-FU)  **speech/swallowing** | BFMDRS (BL, 4y- and LT-FU)  **neck** | BFMDRS (BL, 4y- and LT-FU)  **upper extremities** | BFMDRS (BL, 4y- and LT-FU)  **trunk** | BFMDRS (BL, 4y- and LT-FU)  **lower extremities** |
| --- | --- | --- | --- | --- | --- | --- | --- |
| 1 | 0/1/0 | 6/0/0 | 4/0/0 | 8/2/0 | 13/6/0 | 12/0/0 | 2/0/4 |
| 2 | 0.5/0/0 | 4.5/0.5/1 | 1/1/0 | 8/0.5/0 | 12/1/2 | 0/0/0 | 0/0/0 |
| 3 | 6/0/0 | 4/0/0.5 | 0/0/0 | 6/0/0 | 20/0/4 | 8/0/0 | 0/0/0 |
| 4 | 2/0/0 | 6/2/0.5 | 8/4/0 | 8/0/0 | 8/4/2 | 0/1/0 | 2/2/0 |
| 5 | 3/2/0 | 8/2/0.5 | 8/1/0 | 2/0/0 | 5/0/0 | 0/0/0 | 12/2/2 |
| 6 | 0/4/2 | 4/0.5/0.5 | 0/0/0 | 4/2/2 | 0/0/0 | 8/0/0 | 8/0/0 |
| 7 | 6/1/2 | 6/0/0 | 5/0/0 | 4/0/0 | 16/0/0 | 6/0/0 | 12/0/0 |
| Mean ± SD | 2.8±2.7; 1±1.5; 0.6±1.0 | 5.7±1.5; 0.7±0.9; 0.4±0.3 | 3.7±3.5; 0.9±1.5; 0±0 | 5.7±2.4; 0.6±1.0; 0.3±0.8 | 10.6±6.8; 1.6±2.4; 1.1±1.6 | 4.9±4.9; 0.1±0.4; 0±0 | 5.1±5.4; 0.6±1.0; 0.9±1.6 |

**Suppl. Table 2:** Individual patient subscores of the Burke–Fahn–Marsden Dystonia Scale for motor impairment at BL, 4y-FU and LT-FU, respectively.
